# Evaluating the Impact of Improving Access on Consumption of Fruits and Vegetables in a Rural Community in Texas: A Modeling Study

**DOI:** 10.1089/heq.2018.0090

**Published:** 2019-07-25

**Authors:** Nicole D. Katapodis, Donglan Zhang, Philippe J. Giabbanelli, Yan Li, Conrad P. Lyford, Janani Rajbhandari-Thapa

**Affiliations:** ^1^ Department of Health Policy and Management, University of Georgia, Athens, Georgia.; ^2^ Computer Science Department, Furman University, Greenville, South Carolina.; ^3^ Center for Health Innovation, the New York Academy of Medicine, New York, New York.; ^4^ Department of Population Health Science and Policy, Icahn School of Medicine at Mount Sinai, New York, New York.; ^5^ Agricultural and Applied Economics, Texas Tech University, Lubbock, Texas.

**Keywords:** modeling, rural, fruit and vegetables, diet, chronic diseases

## Abstract

**Purpose:** Most residents in rural regions of the United States consume fewer amounts of fruits and vegetables (FVs) compared with their urban counterparts. Difficulties in access to FVs often contribute to different consumption patterns in rural regions, aside from a lack of education or motivation for eating healthy foods. This article uses simulation methods to estimate the relationship between increasing food access and FV consumption levels in a targeted rural community.

**Methods:** An agent-based model previously developed to predict individual dietary behaviors was used. We adapted it to a rural community in west Texas following a two-step process. First, we validated the model with observed data. Second, we simulated the impact of increasing access on FV consumption. We estimated model parameters from the 2010 census and other sources.

**Results:** We found that decreasing the driving distance to FV outlets would increase FV consumption in the community. For example, a one-mile decrease in driving distance to the nearest FV store could lead to an 8.9% increase in FV consumption; a five-mile decrease in driving distance could lead to a 25% increase in FV consumption in the community. We found that the highest marginal increase in FV consumption was when the driving distance decreased from 3.5 miles to 3 miles.

**Conclusions:** Analysis to inform policy alternatives is a challenge in rural settings due to lack of data. This study highlights the potential of simulation modeling to inform and analyze policy alternatives in settings with scarce data. The findings from modeling can be used to evaluate alternative policies in addressing chronic diseases through dietary interventions in rural regions.

## Introduction

A diet rich in fruits and vegetables (FVs) can be a protective factor for many chronic diseases, including obesity, hypertension, diabetes, and cardiovascular disease.^1^ However, research has shown that most people in the United States do not consume enough FVs; only about 15.1% of women and 9.2% of men report eating the recommended amount (five servings or more) of FVs.^2^ Furthermore, over the past 7 years, per capita FV consumption has declined in the United Sates.^3^ As part of the push to increase FV consumption, the Healthy People 2020 introduced several nutritional objectives that recommend Americans increase their FV consumption.^4^ Another key mission of the Healthy People 2020 goal is to identify population experiencing health inequalities with the ultimate goal of eliminating health disparities, including rural disparities. Rural populations often have higher rates of chronic disease and poorer overall health due to greater prevalence of risk factors, including smoking, physical inactivity, and unhealthy dietary behaviors.^4^ Specifically, there exists a disparity in FV consumption between rural and urban residents; rural adults are less likely to consume five or more servings of FVs per day.^5^ Fresh produce is the golden standard, but research shows nutrients vary in all sources of FVs. Some vitamins and minerals are higher in fresh produce, while other are better maintained in frozen, canned, or dried products, which can increase their shelf life.^6^ However, access to fresh produce is one of the most influential factors that contribute to significant differences in FV consumption across different rural neighborhoods.^5^

Texas has a striking rural–urban disparity in health and ranks in top five states with highest rates of rural uninsured populations.^7^ The daily consumption of FVs, an important healthy lifestyle measure, among residents in the state of Texas is low.^2^ The state has adopted several programs and initiatives to increase access to FVs (e.g., Growing and Nourishing Healthy Communities and Farm to Work). However, there is a need to identify alternative policies that may be effective or more effective at addressing the low FV consumption concerns in targeted rural communities where many residents live and work on farms growing FVs.^8^ Furthermore, literature has shown that simply building new supermarkets to increase FV consumption is not enough.^9,10^ Our study uses the computational technique of agent-based modeling (ABM) increasingly used in the public health field.^11–13^ The *National Nutrition Research Roadmap* called for the use of ABM in nutrition research to advance exploration of the impact of multiple interventions.^14^ In the past, ABM has been used to address food system issues by providing a method to test the effects of alternative policies on complex systems.^15–18^

This study combines available local data with an ABM approach to generate predictions on the response of increasing FV accessibility on FV consumption in a rural community in Texas. Our objective is to provide new insights on how to increase consumption of FVs by considering population demographics, food choices, local food environments, interventions, and social norms in a rural context.

## Methods

### Model overview

ABM is a technique that tracks every individual in the system. We thus model individuals rather than aggregated groups. This approach allows us to represent the heterogeneity of our target population. Specifically, our simulated individuals differed by their demographic characteristics (e.g., age, gender, and educational attainment) and health beliefs (whether a person strongly prefers healthy foods). The model did not include income because in general, there was a close link between education and income—those with a higher education earn a higher income.^19^ We captured income effect by the price sensitivity parameter in the model, and the model focuses on nutrition beliefs/mindsets, which are tied more to education than income.^20^ While other individual-level techniques exist (e.g., cellular automata), ABM captures interactions between individuals and interactions between individuals and their environment. This is essential to represent how social norms conveyed by peers can shape an individual's health beliefs (i.e., effects of the social network) and to model accessibility. In rural areas, FV access is one of the most important factors driving FV consumption.^21^ Hence, in this first study from an ongoing project, we modeled the impact of accessibility on rural residents' FV consumption, while holding other factors constant in the model.

We previously developed one of the most detailed ABM approaches to predict dietary behaviors, considering the joint effects of individual- and neighborhood-level factors (e.g., age, gender, education, social network, and the food environment). We applied it to examine the impact of policies on food consumption in urban populations in California^17^ and later in New York City.^18^ The model is based on the multilevel theory of population health that underscores the role of cognitive habits in human behaviors.^22^ According to this theory, peers, cognitive habits related to dietary behaviors, income level, access to healthy or less healthy food retailers, and food prices influence individual decision-making regarding daily food choices. Model parameters and assumptions have been presented in a previous study^17^ (for more information on data sources for model parameters, agents' decision-making equations, and model calibration, please find an appendix available as a supplement to the online version of this article by Zhang *et al.*^17^). The present study is the first to bring this detailed model into a rural population. When applying the urban model to the targeted rural population, we tweaked the parameters on population profiles and community environment, including land areas, and number of healthy and unhealthy food outlets, while we kept the parameters measuring individual attitudes and behaviors the same as in the original urban model.^23^ We used the Java programming language to program the ABM.

### Model calibration

We first placed simulated individuals in the targeted rural community. The food choices of simulated individuals were empirically calibrated through a probability function, which accounts for sociodemographic characteristics, health beliefs, food prices, price sensitivity, and accessibility to different food outlets (e.g., supermarkets, fruit and vegetable markets, and limited and full-service restaurants). The relative influence of each factor on the food choice is represented by a weight in the model whereby each individual values these factors differently based on sociodemographic characteristics. For instance, an individual with a low education level puts a higher weight on food prices than on other factors. In addition, an individual with peers in the same social network who prefer healthy eating values healthiness to other factors (i.e., taste, access, and price) in food choices. Therefore, changing one factor in the model (i.e., access to fresh produce) will not necessarily lead to a change in food choices, and the effect will vary according to the sociodemographic difference of the individuals. Food accessibility was measured by whether an individual could drive to a given type of food outlet within a predefined driving distance. As documented in previous studies, the majority of rural residents drive to food outlets, only 4% of rural residents walk to grocery stores compared with 20% of residents in urban areas.^24^ While rural residents generally have higher vehicle ownership, those who lack reliable access to personal vehicles are particularly isolated given the longer distance to stores and the lack of public transportation options.^24^

Since the model was originally created for an urban area, the data used to calibrate the model to a rural setting are presented in Table 1, which includes a population per square mile of 1503.4. The average driving distance to the nearest food outlet in the original model was set at 1 mile. However, this does not hold true for our rural community. The neighborhoods in rural Texas live 6.7 miles away from the nearest food store containing FVs.^24^ As such, we calibrated the driving distance for a rural region such that 1 mile in the simulation model reflects around 5 miles in rural Texas. The model output is the percentage of the population consuming more than two servings of FVs per day.

**Table 1.** Population Characteristics and Food Environment in the Urban Community (Urban Model) in California and the Rural Community (Rural Model) in West Texas

Community characteristics	Urban model	Rural model
Land area, square miles	22.97	3.43
Population per square mile	5969.6	1503.4
Age (%) 18–65	54.7	54.7
Female (%)	51.9	50
Education		
High school graduate or higher	27.9	66.4
Bachelors degree or higher	61.2	8
Food environment characteristics		
Limited-service/fast-food restaurants	87	3
Full-service restaurants	26	4
Supermarkets	2	2
Fruit and vegetable markets	14	1
Accessibility radius (miles)	1	5
Fresh vegetable price index	0.72	0.72
Fresh fruit price index	2.71	2.71
Social influencability index	0	0
Ratio (unhealthy food outlets/healthy food outlets)	7	2

Data on population characteristics were obtained from the 2010 Census. Data on the food environment were obtained from the Food Environment Atlas and previous literature. FV price index and FF price index were derived from the study by Powell et al.,^26^ who estimated price index with data from the American Camber of Commerce Research Association.

FV, fresh vegetable; FF, fresh fruit.

### Model validation

Due to lack of data from rural areas, it is difficult to conduct an empirical-driven policy analysis to inform localized decision-making. We chose this rural community because we took advantage of a prior intervention study^25^ that had collected detailed FV consumption data to showcase the potential of using simulation modeling for policy analysis in rural areas where limited or no data are available. We estimated a baseline scenario and compared the simulated result with the observed data^26^ from the rural community for model validation.

### Simulation of increasing FV access on FV consumption

We then developed several hypothetical scenarios of decreased driving distance to the FV store and simulated the effect of potential policy interventions designed to increase the accessibility of FVs. This simulation would allow us to assess (1) the effect of increasing consumer accessibility—shortening the driving distance to the nearest FV food store—on FV consumption and (2) the nature of the relationship between accessibility and consumption decisions by considering the complex interplay of other individual and environmental variables.

To shorten the driving distance to the nearest FV store, our hypothetical interventions represented real policy scenarios such as to increase the density of FV stores in the community or to bring the FV stores closer to consumers.^9^ We predicted the impact of these hypothetical interventions by decreasing the driving distances on the proportion of the population who consume two or more servings of FVs daily.

## Results

**FIG. 1.**
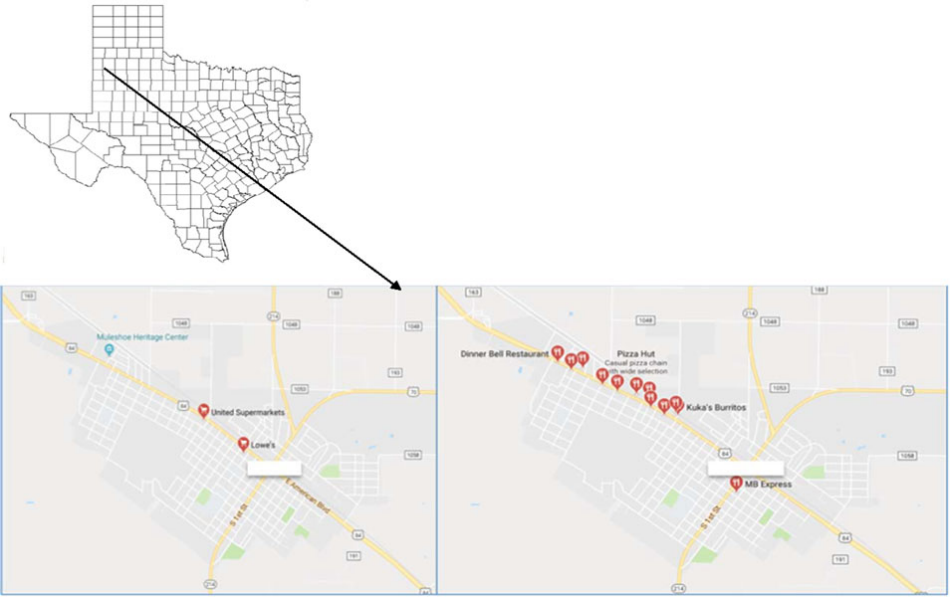
Map of the rural community in the State of Texas and restaurants (*right*) and supermarkets (*left*) in the rural community. (Source: Google maps)

Figure 1 includes a map of the rural community in west Texas and shows the availability of restaurants and supermarkets in the community. Table 1 presents data on the population demographics and the food environment in the original urban community as well as the present, targeted rural community. We estimated input variables for the model from multiple data sources, including the 2010 US Census, Department of Agriculture's Food Environment Atlas, and previous literature.^26^ The 2010 Census measured a variety of factors, including population demographics and socioeconomic status, of the modeled rural community.^27^ As noted in Table 1, the community has a low population density, equal gender distribution, and the majority of residents are adults of working age.

**FIG. 2.**
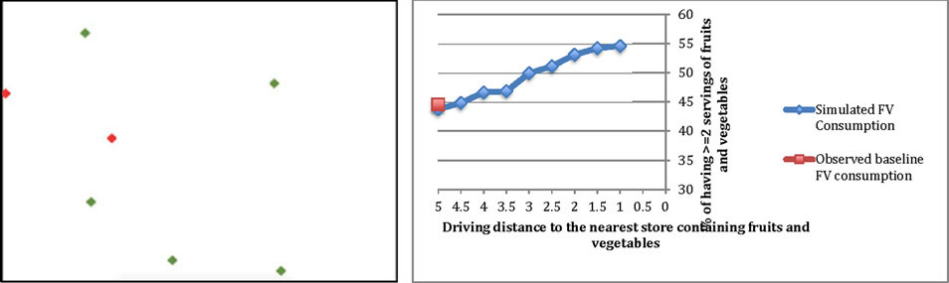
Agent-Based Simulation Model Output: fruit and vegetable consumption (*right*)^1^ and food distribution map (*left*).^2^ ^1^The percentage of residents consuming two or more fruits and vegetables per day in a rural community in west Texas. Results are presented as population means. ^2^*Green dots* represent healthy food outlets. *Red dots* represent unhealthy food outlets in 3.43 square miles.

Figure 2 (left) depicts a food distribution map generated by the model. Green dots represent food retailers selling FVs and red dots represent retailers that do not. It shows that food retailers in the rural community are geographically dispersed. Figure 2 (right) shows the response curve of the percentage of the population consuming two or more servings of FVs at baseline and under several policy scenarios. The simulated consumption level at baseline was that 43.7% of the population consumes at least two servings of FVs per day. This figure was close to the 44.5% observed from the rural community survey in 2012. We found that consumers' consumption of FVs was sensitive to access to FV food stores. Decrease in distance to the FV store would increase consumption of FVs in the community. The results show that a one-mile decrease in driving distance to the nearest FV store could lead to an 8.9% increase in FV consumption. Furthermore, a five-mile decrease in driving distance could lead to a 25% increase in FV consumption.

Furthermore, results in Figure 2 (right) show the response curve of changes in FV consumption with decreasing driving distance. Results show that the rate of increase in FV consumption with each unit decrease in driving distance is diminishing. The highest marginal effect (e.g., positive marginal effect) in FV consumption was found when the driving distance was decreased from 3.5 miles to 3 miles. This suggests that interventions should focus on increasing store outlets within a 3-mile driving distance in rural settings.

## Discussion

We used ABM of dietary behaviors to evaluate the impact of policies that would improve access to FVs in a rural community. ABM can help us take a holistic view of human behaviors and understand how different factors at multiple levels can influence dietary behaviors based on existing data. We chose to focus on accessibility to fresh FVs as disparities in food access are greatest in rural communities. These disparities most often arise from the distance and methods of transportation involved in access.^28^ We found that policies that improve access to FVs, such as bringing FV retailers closer to community residents or increasing the density of FVs in a rural community, could substantially affect their consumption. In comparison, a study conducted in rural counties in Texas found that each additional mile traveled to the supermarket was associated with a three percentage point decline in the probability of consuming two or more servings of fruits per day and a 1.8 percentage point decline in the probability of consuming three or more servings of vegetables per day.^24^ Furthermore, mothers participating in the Women, Infants, and Children (WIC) program in rural West Virginia identified long distance and travel time as factors compromising their ability to get to food sources.^29^

In addition, this study also shows that the highest marginal effect (the change in proportion of people consuming at least two servings of FVs) was when the driving distance decreased from 3.5 to 3 miles. The marginal effect increases with each unit decrease in driving distance between 4–3.5 and 3.5–3 miles. This finding is very relevant as interventions and policies are based on competing means and resources. Previous interventions targeting increased FV access in rural areas have focused on improving nutrition in school lunch programs and the implementation/establishment of home gardens.^30^ It is best to use resources where the rate of return is the highest. This is also another advantage of modeling to analyze policy alternatives before actual implementation for the highest return on investment. Previous research exists on urban form and its influence on grocery shopping and issues of access in urban sites.^31,32^ To the best of our knowledge, the present study represents the first to model healthy eating and local food policies in a rural community. Compared with previous modeling studies in urban regions, we identify that access to FVs affects healthy eating more in rural than urban communities.^17,18^

To reduce urban–rural disparities in health, our results highlight the usefulness of modeling during the design of localized interventions to incorporate the effect of population characteristics and the food environment in studying potential program effectiveness especially in resource-constrained areas. A modeling approach such as the one undertaken here can provide two advantages. First, it provides a framework to relate the program design to its outcome for planning and evaluation purposes, thus bringing a data science approach to decision-making in population health. Second, some policies/interventions may be more effective and/or cost-effective, thus a modeling approach provides a virtual environment in which different program designs can be contrasted to identify the best candidates.^17^ The main contribution of this work is to inform policy decision-making in rural areas considering both rural geography and individual–environment interactions. This approach can evaluate the efficacy of different potential policy and outcome changes in other rural areas and tribal communities. For example, modeling influence of expanding nonretail outlets, as was identified as an alternate source of healthy foods by rural Latino communities in central California,^33^ and modeling impacts of healthy retail interventions among Native Americans with high rates of obesity^34^ living in areas with varying healthy food availability.^35^

Although the interventions examined in this study are hypothetical, they have real-world implications and are consistent with program efforts. Given the difficulty of behavior change, a new forefront in chronic disease prevention and management has evolved where individual-level behaviors are complemented by population- and policy-level changes to mitigate chronic disease risk factors.^32^ The Texas Department of State Health Services has implemented many innovative interventions to improve access to FVs. Examples include Growing and Nourishing Healthy Communities (increasing the availability of FVs through community gardens) and Farm to Work (local farmers delivering FVs to work places).^8^ A concept mapping study found many nonindustry-driven, supply-side policy options to improve access to FVs in rural communities. Such policies include farmers, growing produce, having equitable access to subsidies, crop insurance, agricultural loans, and technical assistance. Additional policies include funding to increase access to transportation that links families to supermarkets and affordable food outlets; bringing markets, produce trucks, farm stands, and food carts closer to accessible locations; and building infrastructure that allows for safe and economically feasible transport of goods to rural markets and consumers.^33^ On the demand side, USDA supports promotion of farmers markets and direct-to-consumer markets with the Supplemental Nutrition Assistance Program (SNAP) and WIC program to improve access to FVs.^36^ This is particularly important in the context of declining SNAP and WIC participation^37^ and underutilization of the cash value voucher for FVs within the WIC program.^38^ Methods such as ABM can provide important insights into the differential impact of interventions on dietary behaviors across different populations and policy scenarios.

Agent-based simulation modeling has tremendous potential to transform the design, planning, implementation, and evaluation of community-level interventions. The use of modeling in this realm is still relatively new, but has been used in community-based obesity interventions.^39,40^ The early work in this field focused mostly on conceptualization of ABM and ways to use it to shape the view of public health systems.^41^ Not until recently, a greater number of modeling studies incorporated empirical data and behavioral theories, which allowed them to be applied to tackle real-world problems.^42,43^ Our study, based on an empirical-driven model, tries to elucidate the mechanism behind people's food choices and fills a research gap in understanding the interaction between the environment and individual behavior in settings where data are sparse. As the use of simulation modeling to guide such community-level interventions grows, public health practitioners and policy makers will become more informed using this method. The use of this method will advance as more data become available and high computational capacity becomes possible.

Our study has several limitations. First, all models are constructed based on a boundary: certain features of the real world will be included (e.g., because previous research highlights their importance and/or due to data availability) and others will not. As all models, our ABM is thus a simplification of the real world and includes assumptions that should be understood before interpreting results. Furthermore, we investigated a single variable, which is accessibility to FVs. In the future, we will look at the impact of changes in social networks and nutrition mindsets influenced by social networks in the rural population as these factors likely differ in rural compared with urban populations. For example, we assumed that peers within the community only influence health beliefs without considering social networks outside of the community. Despite these assumptions and simplifications, our model provides a reasonable representation of the real world, as demonstrated by satisfactory model validation results. Second, given that the model was originally created for an urban region, there are limitations regarding the geographical size of rural regions. For example, rural communities are loosely connected and residents may be more independent and isolated, thus community social connections may have a smaller effect on cognitive habits and food behaviors. Third, some datasets (e.g., 2010 Census) used to estimate model parameters are relatively old. Model parameters should be updated once more recent data become available. Finally, although model outcomes were validated with baseline outcomes, we do not have data related to real-world interventions for model validation. Ideally, a model could be continuously calibrated as data from the intervention in their real-world counterpart become available. A manageable approach requiring minimal technology is to regularly update the model using a participatory modeling approach.^17,39,44,45^

Most residents in rural regions of the United States do not consume the comparable amount of FVs per day as their urban counterparts. Difficulties with access to fresh FVs contribute to different consumption patterns in rural regions. A system approach should be adopted to understand the complex relationship among these factors. Simulation models can provide policymakers with a useful tool to evaluate the impact of policy interventions before their implementation. ABM of dietary behaviors that accounts for a rural region's geography has the potential to inform the local community on how increasing access to FVs increases consumption of FVs.

## Abbreviations Used

ABM: agent-based modeling
FF: fresh fruit
FVs: fruits and vegetables
SNAP: Supplemental Nutrition Assistance Program
WIC: Women, Infants, and Children
